# Sonochemical Degradation of Benzothiophene (BT) in Deionized Water, Natural Water and Sea Water

**DOI:** 10.3390/molecules24020257

**Published:** 2019-01-11

**Authors:** Khadijah M. Al-Zaydi, Christian Petrier, Sameera M. M. Mousally, Sana T. Arab, Moamen S. Refat

**Affiliations:** 1 Department of Chemistry, Sciences Faculty -AL Faisaliah, King Abdulaziz University, Jeddah, P.O. Box 50918, Jeddah 21533, Saudi Arabia; Christian.Petrier@univ-savoie.fr (C.P.); smousally@kau.edu.sa (S.M.M.M.); dr.s.arab@hotmail.com (S.T.A.); 2 Chemistry Department, Faculty of Science, University of Jeddah, P.O. Box 80327, Jeddah 21589, Saudi Arabia; 3 Rheology and processes Laboratory UMR 5520- UJF-Grenoble INP-CNR, Laboratoire Rhéologie et Procédés, Domaine Universitaire, 38041 Grenoble, France; 4 Chemistry Department, Faculty of Science, Taif University, P.O. Box 888, Al-Hawiah, Taif 21974, Saudi Arabia; msrefat@yahoo.com; 5 Department of Chemistry, Faculty of Science, Port Said, Port Said University, Port Said 42526, Egypt

**Keywords:** benzothiophene, sonochemical degradation, water treatment, advanced oxidation processes

## Abstract

This paper deals with the sonochemical water treatment of polycyclic aromatic sulfur hydrocarbons (PASHs), one of the most common impurities found in waste water coming from petroleum industry. The best fit of the experimental data appears to be the kinetic parameters determined using the Michaelis-Mentonmodel in the concentrations range of the study. For the initial increase in the degradation rates, it is simply considered that the more the bulk concentration increases, the more the concentration in the interfacial region increases. This will be explained by Michaelis-Menton kinetics. The influence of organic compounds in the water matrix as a mixture with Benzothiophene (BT) was also evaluated. The results indicated that BT degradation is unaffected by the presence of bisphenol A (BPA). Finally, the results indicated that ultrasonic action is involved in oxidation rather than pyrolitic processing in the BT sonochemical degradation.

## 1. Introduction

Polycyclic aromatic sulfur hydrocarbons (PASHs) are compounds found as impurities in crude oil. PASHs can be found in waste water from the petroleum industry. These sulfur compounds accumulate in sediments and animal tissues, exhibiting toxic and mutagenic properties [1,2].

Classical treatment used in waste water and drinking water plants cannot always supply water that meets current and future regulation requirements. Therefore, since the early 1970s, processes named Advanced Oxidative Processes (AOPs) have been under development to improve the efficiency of water treatment [3]. AOPs are based on the production and the use of the hydroxyl radical °OH. Often, this powerful chemical entity reacts with organic and mineral compounds at a rate only limited by diffusion. This was evidenced formerly in Fenton’s reaction (Equation (1)).

(1) $$H_{2}O_{2}+{\mathrm{Fe}}^{2+}\;⟶\;{\mathrm{HO}}^{{}^{\circ}}+{\mathrm{OH}}^{-}+{\mathrm{Fe}}^{3+}$$

It can be also produced in different ways: UV irradiation of water, electrochemically, ozone decomposition in alkaline water, hydrogen peroxide and ozone photodecomposition and photocatalys is at semiconductor’s surface [4].

Since 1929, it was established that propagation of an ultrasonic wave in aqueous medium can be conducive to oxidation processes. Further, as postulation of the involvement of hydroxyl radicals was put forward, the technique was evaluated for the elimination of organic pollutants in water [5,6].

In the ultrasonic method, hydroxyl radical generation does not come from a direct interaction between the acoustic wave and the matter like what occurs in photochemistry. They are produced upon the pulsation and collapse of cavitation bubbles that are created upon the pressure change caused by the propagation of the wave in the liquid [7]. The collapse of the bubble occurs adiabatically, generating high pressure (200–400 atm) and high temperature (4000 °C) in a µs scale [8]. At this temperature vaporized water and molecular oxygen dissociate, releasing the °OH radical into the medium (Equation (2)) [9]. The radicals formed in this reaction are highly reactive and interact rapidly with other radical or chemical species in solution. When the ultrasound is applied, it will induce the sonolysis of water molecules and the thermal dissociation of any oxygen molecules present, to produce different kinds of reactive species such as OH, H, O and OOH. Reactive-species production ensues by way of the following reactions, with ultrasound denoting the ultrasonic irradiation [5,6,7,8]. Sonolysis of water also produces H_2_O_2_ and H_2_ gas via OH and H. Though oxygen enhances sonochemical activity, its presence is not essential for water sonolysis, as sonochemical oxidation and reduction processes can proceed in the presence of any gas [9]. However, the presence of oxygen also allows the H-forming OOH to be scavenged, with this acting as an oxidizing agent. The hydroxyl radicals generated during cavitation can be used in the oxidative degradation of organic pollutants in an aqueous system. The sonochemical destruction of pollutants in the aqueous phase generally involves several reaction pathways, such as pyrolysis inside the bubble and hydroxyl radical-mediated reactions at the bubble–liquid interface and/or in the liquid bulk. The extreme temperature conditions generated by a collapsing bubble can also lead to the formation of radical chemical species [10]. Then bubble of cavitation that generates °OH is connected to the AOPs.

(2) $$\begin{array}{l} H_{2}O⟶{\mathrm{HO}}^{{}^{\circ}}+H\\ O_{2}⟶2O\\ O+H_{2}O⟶2{\mathrm{HO}}^{{}^{\circ}}\\ H+O_{2}⟶{\mathrm{HO}}^{{}^{\circ}}+O \end{array}$$

To summarize, in water, the bubble of cavitation can be considered as a microreactor that incinerates volatile molecules inside the bubble and oxidizes structures with low fugacity character at the interface area of the bubble [10].

In light of the serious problem of PASHs that cannot be efficiently eliminated with classical activated sludge processes, many research articles are devoted to sonochemical degradation of organic contaminants of water, however few of them report the elimination of aromatic sulfur compounds [11,12,13].

Therefore, herein, the model compound studied for the evaluation and optimization of degradation rates in aqueous media for organic sulfur compounds contaminants of soils and waters using ultrasound action was benzothiophene (BT).

## 2. Materials and Methods

### 2.1. Reagents

Benzothiophene (BT) (molecular formula C_8_H_6_S, FW 134.20 g/mol) was obtained from Sigma Co. Bisphenol A/Diphenylolpropane (BPA) (molecular formula C_15_H_16_, FW 228.29 g/mol) was obtained from Sigma Co.

### 2.2. WaterSamples

- Deionized water: Prepared with activated carbon and ion exchange resins from Fisher Bioblock Scientific. Conductivity <1.5 µS.
- Natural water: Salts concentration = 2.078g/L, conductivity = 2.15 mS, Ca^2+^ = 468 mg/L, Mg^2+^ = 74.5 mg/L, Na^+^ = 9.4 mg/L, SO_4_^2−^ = 1.121 mg/L and HCO_3_^−^= 372 mg/L.
- Sea water: Prepared from sea salts mixture from Sigma Co.: Salts concentration = 30 g/L, conductivity = 33.4 mS, Cl^−^ = 15239 mg/L, Na^+^ = 8516 mg/L, SO_4_^2−^ = 2101 mg/L, K^+^ = 332 mg/L, Ca^2+^ = 316 mg/L, HCO_3_^−^ = 158 mg/L, Sr^2+^ = 6.9 mg/L, B^+^ = 4.4 mg/L and Br^−^ = 44.2 mg/L.

### 2.3. Sonochemical Equipment

**Figure 1:**
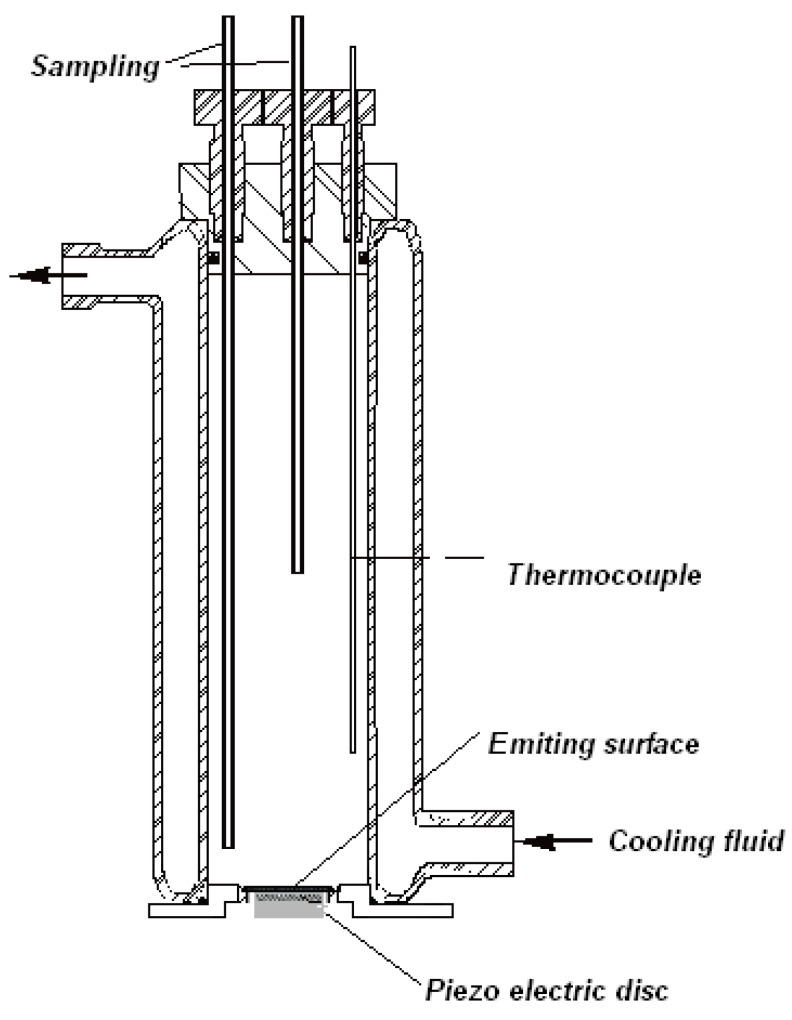
The general scheme of a sonochemical reactor operating in the high frequency range (200–2000 kHz).

Sonochemical reactions were conducted using Sea and Sun technology equipment consisting of a T&C amplifier connected to a RG transducer. The amplifier can operate in the range of 3 Hz to 1 MHz with a power output in the range 0–200 W. Figure 1 represents the general scheme for the sonochemical reactor used. This reactor was calibrated according to previously reported literature [14]. All of the experiments were conducted in the sonochemical reactor depicted in Figure 1. The volume treated was 300 mL. The water circulating jacket was connected to a thermostated bath to set the temperature of the liquid inside the reactor at 21 °C ± 1 °C. Temperature was checked with the help of a thermocouple. The ultrasonic generator was set at 352 kHz for an electric output power of 80W. At the base of glass cylinder, a lead titanatezirconate piezoelectric ceramic (Quartz et Silice P762, Nemours, France), 500 kHz, diameter 4 cm) was fixed at the center of the stainless-steel disc.

### 2.4. Reactor Calibration

The cavitation intensity is defined by many conditions, which include the following: The liquid nature, the temperature, the pressure and the dissolved gas linked to the pressure amplitude (Pa) of the wave generated in the liquid by the vibrating surface. The pressure is related to the acoustic power (Pw) and the amount of energy delivered in the medium each second per unit of volume [Pa = (2ρPw)^1/2^] (ρ = density of the liquid) [15]. According to the size of the reactor, its shape and the transducer used to conduct the experiments and multiple reflections at the surfaces, the Pa and Pw values cannot be the same at each point of volume subjected to ultrasonic action [16,17]. To overcome this problem and to globally determine the available power, the calorimetric method is commonly employed. In this approach, it is assumed that the absorbed ultrasonic power is converted into heat. Then, P_th_ = mCp(dT/dt) {P_th_ = calorimetric power in watts, m = mass of the liquid, Cp = heat capacity of the liquid, T = temperature of the liquid and t = time} [18,19]. A more complete description of the energy conversion requires the measurement of the input electric power needed to set the transducer in motion. This can be done using a classical watt meter (Pe), and it will provide an indication of the efficiency of the energy transfer. For a tuned and designed transducer, the efficiency in the system (P_th_/Pe) is 45–70% [16,20].

**Figure 2:**
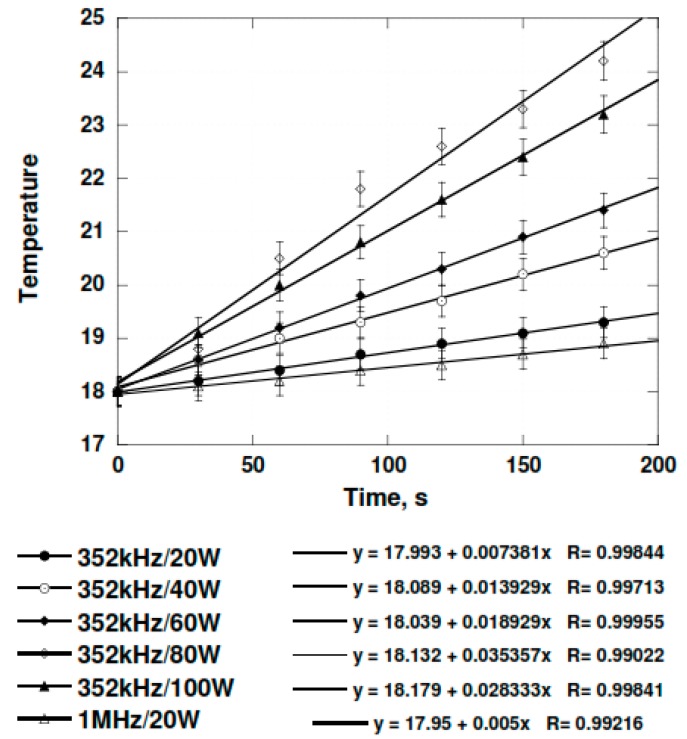
Evolution of the temperature in the sonochemical reactor containing water (400 mL).

Calorimetric calibration of the sonochemical equipment at 352 kHz and 1052 kHz was conducted with 400 mL of water in the reactor. The average increase of the temperature for each electrical input from three separate experimental sets were recorded. The temperature was determined with the help of a thermocouple immersed in the reactor (Figure 2).

The measurements at 352 kHz could be done between 20 and 100 electric watts. It was not possible to work above 100 W due to the fast increase of the reflected electrical energy. At the frequency of 1052 W, only the power of 20 W could be used. The ultrasonic power that truly dissipated in the medium could be determined from the rate of temperature increase at each electrical power from Figure 2.

P_th_ = mCp(dT/dt) (3)

m = 400g, Cp = 4.18 J, dT/dt = rate obtained from Figure 2.

**Figure 3:**
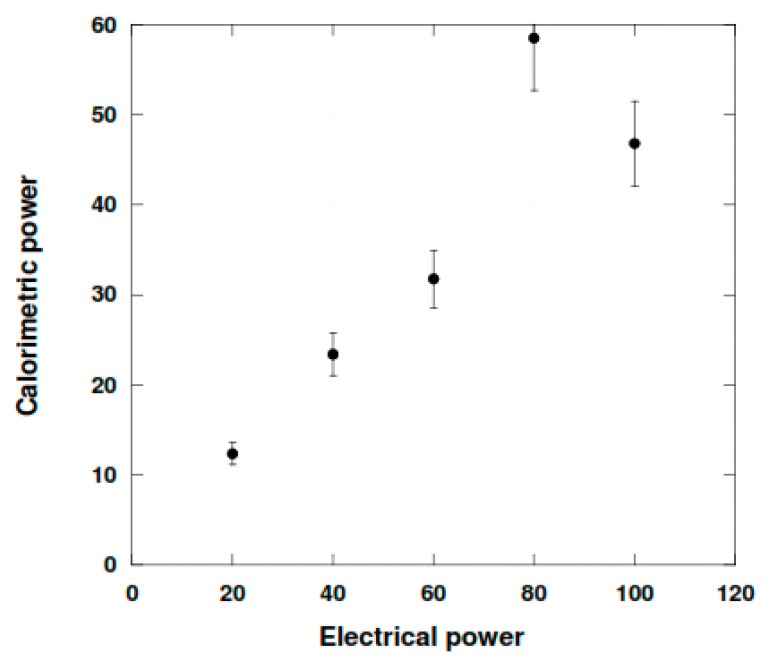
Ultrasonic energy (colorimetric method) absorbed in water with increasing electrical power input 352 kHz; 400 mL.

Figure 3 exhibits the conversion of the electric energy into acoustic energy. It shows that the efficiency of the transfer was equal to 56% till 80 W, a rather good value. Above this value, the conversion was found less efficient. This may be due to the overloading of the transducer.

### 2.5. Analyses

Quantitative analysis of BT was done using a HPLC apparatus consisting of the following: Waters Associates 510 instrument equipped with a Supercoil LC-18 column (ID = 4.6 mm, length = 250 mm) solvents delivery system; Waters 486 absorbance detector set at 190 nm. The mobile phase, water/acetonitrile (4/6) for BT and water/acetonitrile (2/8) for BTP was run in an isocratic mode. Qualitative analysis of the primary products of the reaction was carried out in a GC/MS experiment. The reaction volume (300 mL) was concentrated on a BondElut Jr cartridge for 10 min in the case of BT. After washing the absorbed products with deionized water (5 mL), they were eluted with acetonitrile (2 mL). Mass determination was achieved using the GC/MS Polaris Q equipment connected to a Trace GC mass analyzer (Thermofinnigan).The column was an OPTIMA-5 MS Accent from Macherey-Nagel (0.25 µm × 30 m × 0.25 mm). The temperature of the oven was initially set for 3 min at T = 70 °C. The temperature was then increased to T = 300 °C at the rate of 10 °C/min. The gas used was Helium at a flow rate of 1.7 mL/min.

## 3. Results and Discussions

### 3.1. Benzothiophene (BT) Sonochemical Degradation

#### 3.1.1. Different Water Types effect on Sonochemical Degradation

The initial solution C_o_ that was used for the study was prepared upon BT dissolution (90 mg/L) in deionized water, (92 mg/L) in natural water and (95 mg/L) in sea water.

**Figure 4:**
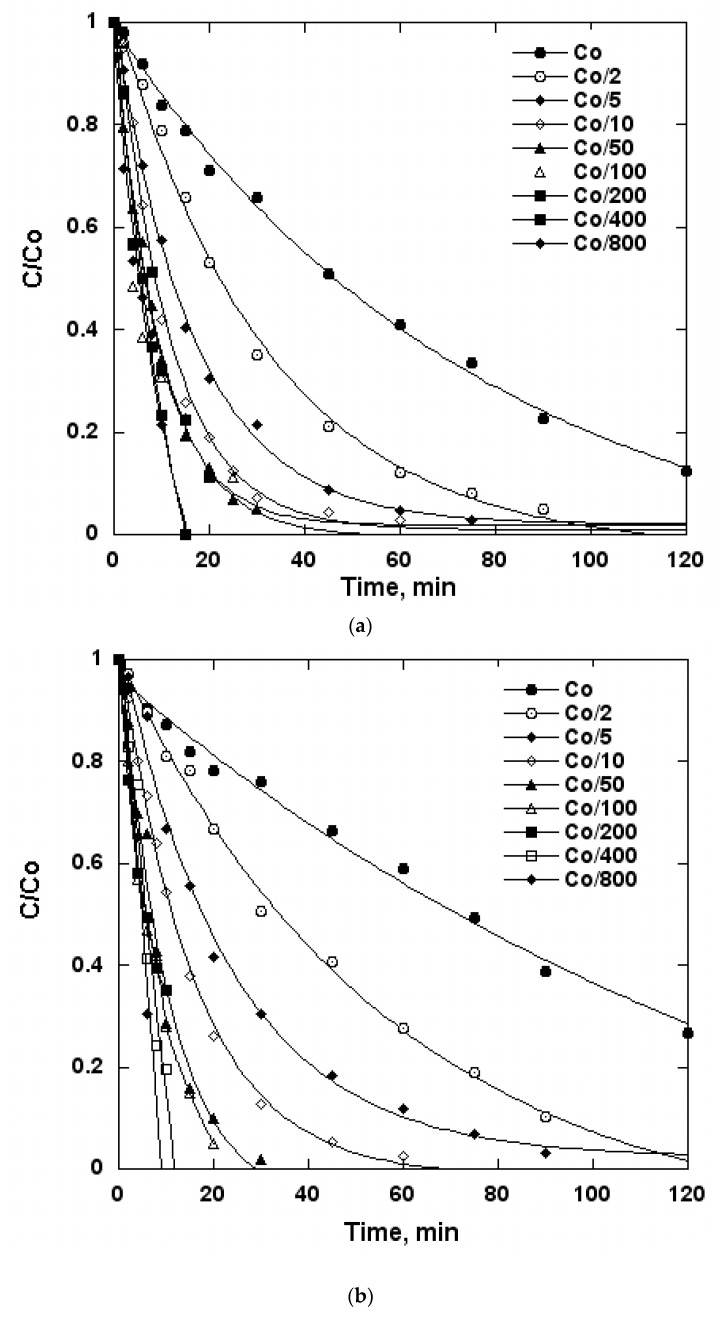

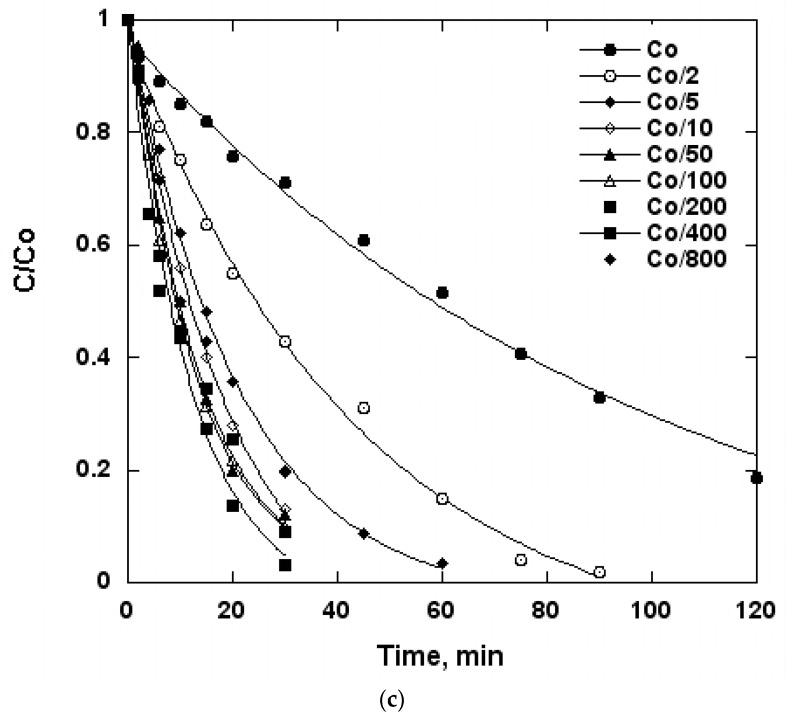
Sonochemical degradation at 352 kHz of benzothiophene (BT) in water (300 mL), temperature = 21 °C ± 1 °C; electrical input = 80 W; (**a**) deionized water C_o_ = 0.67× 10^−3^ ML; (**b**) natural water C_o_ =0.71 × 10^−3^ ML; (**c**) sea water C_o_ = 0.95× 10^−3^ ML.

Figure 4 exhibits the main features of BT degradation upon ultrasound action. It was observed that the time course of the concentration followed an exponential decrease in all cases: Deionized water, natural water and sea water. However, it can be seen that the kinetic cannot be a first order kinetic, especially in the higher concentration range.

The best fit of the experimental data appeared to follow a Michaelis-Menten [21] (using KaleidaGraph software, Synergy Software, 2457 Perkiomen Ave, Reading, PA 19606, USA) model in the concentrations range of the study. R = (11.7 × C)/(155.7 + C) in the case of deionized water, R = (6.86 × C)/(82 + C) in the case of natural water and R = (11.2 × C)/(205 + C) in the case of sea water.

**Figure 5:**
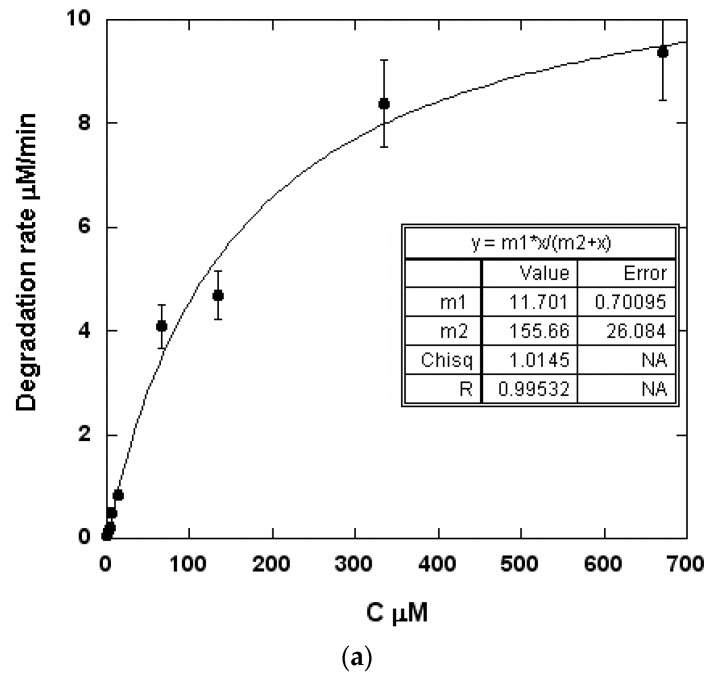

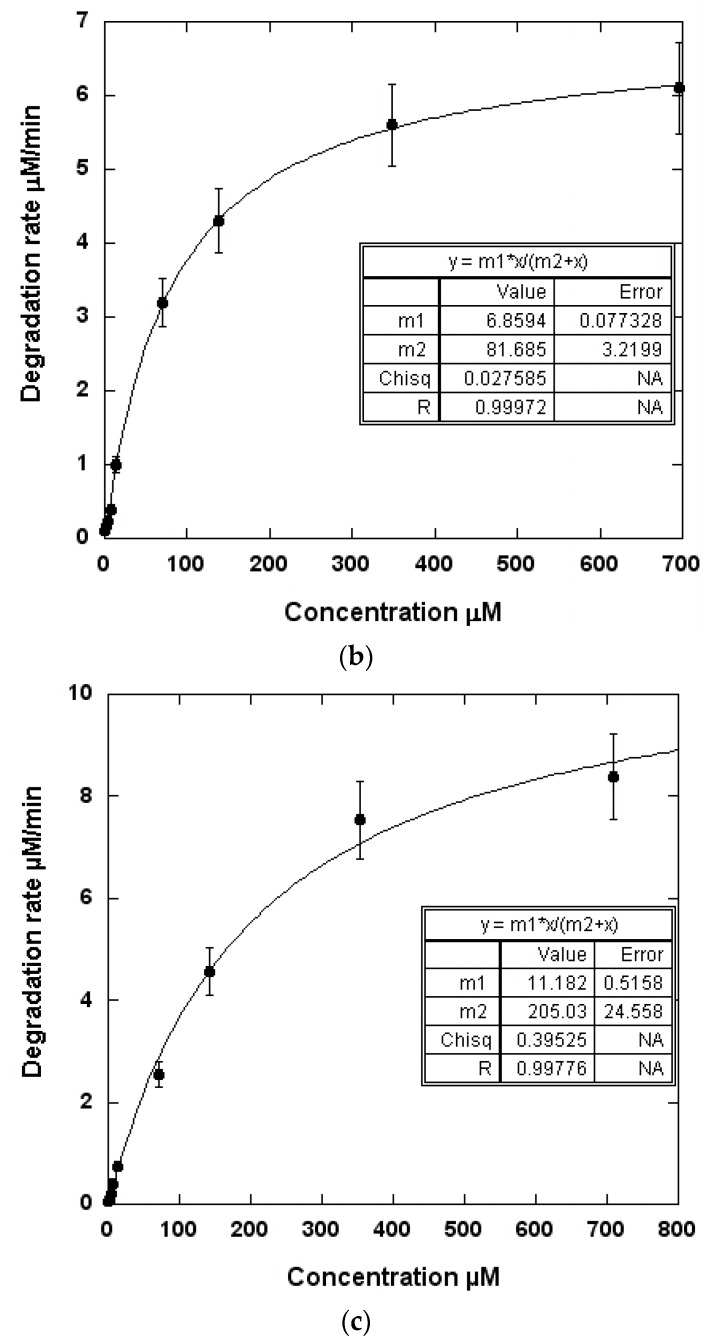
The degradation rates of BT as a function of the concentration(**a**) in deionized water; (**b**) in natural water; (**c**) in sea water (352 kHz; volume = 300 mL; temperature = 21 °C ± 1 °C; electric input = 80 W).

Figure 5 shows the degradation rates of BT as a function of the concentration: 352 kHz; electrical input = 80 W, volume = 300 mL and temperature = 21 °C ± 1 °C.

It was obvious that as the concentration increased, the degradation rate increased until a steady state was reached.

#### 3.1.2. Analysis of the Degradation Products

For this study, analysis was achieved via GC/MS experiments. The most detailed information came from the chromatogram acquire dvia GC/MS using chemical ionization during the detection step. Twelve main products could be evidenced in the case of natural water after 5 min of reaction time (Table 1).

Most of the micro-pollutants present in water possess an aromatic structure, including dyes, drugs, pesticides, and industrial raw organic chemicals. For this reason, a great deal of literature is concerned with the elimination of these types of compounds using ultrasound.

**Figure 6:**
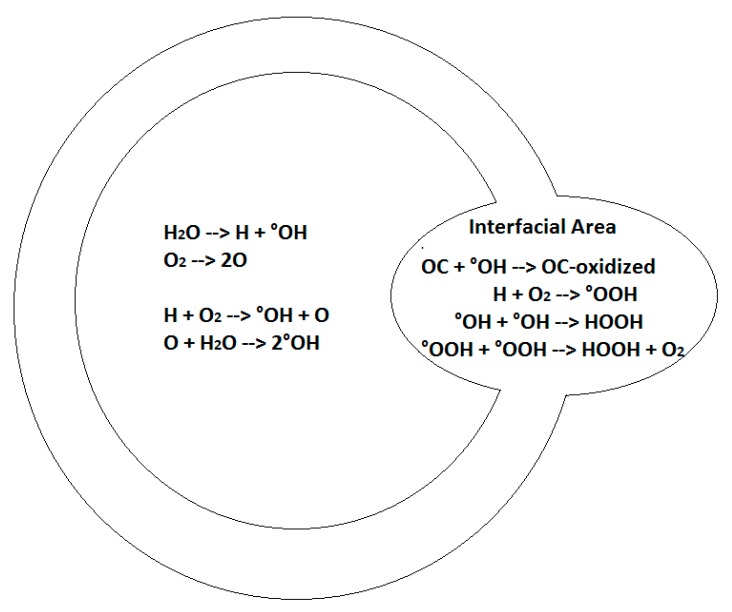
Scheme of the main reactions occurring in and at the interface of the bubble that account for the hydrophilic organic compounds’ (OC) oxidation.

The scheme published by Sehgal and Wang [22] is largely used to approximate the reaction location (Figure 6). Radicals generated in the gaseous phase of the bubble react in a condensed layer at the liquid interface. The nature of this interface is still under discussion [23,24] but it is well established that it accumulates organic molecules depending on their hydrophobic characters [25,26,27]. The more hydrophobic the structure, the greater the accumulation within the interfacial film, the more it reacts with hydroxyl radicals coming from the inside, and the more rapidly degradation occurs [28,29,30].

**Figure 7:**
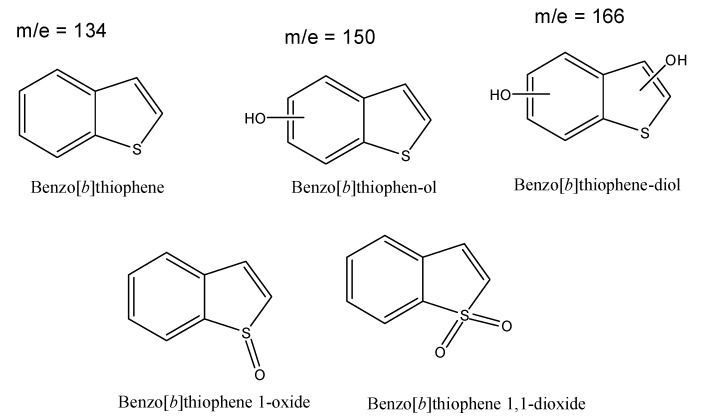
Main products obtain during sonochemical degradation of BT.

Following this scheme, and in agreement with mass spectra analysis, BT sonochemical degradation mainly conducts to the formation hydroxylated products, sulfoxide and sulfone (Figure 7).

### 3.2. Kinetics of BT Degradation

In the case of compounds with a low fugacity property (HL constant < 10^−3^ atm·m^3^·mol), the kinetic description of the pollutant sonochemical elimination usually follows a pseudo-first-order law since the elimination curve decreases exponentially. This is clearly the case for the higher concentrations of BT represented in Figure 4a and Figure 2b,c. This could lead to the determination at each concentration of a rate constant K_d_{r =K_d_(Cs); with r = degradation rate, Cs = substrate concentration}. This rate constant declines with an increase in the substrate concentration as exhibited by the three aqueous medias in Figure 5a and Figure 3b,c. Consequently, the use of the rate constant coming from a first order law kinetic should be considered irrelevant. The most appropriate kinetic model was developed first by Serponeet al. [31]. The reaction rate is described by two regimes: (1) a regime at a lower concentration in which the reaction occurs in the bulk, and (2) a regime at a high concentration in which the sonochemical reactivity occurs at the bubble-liquid interface and connects the sonochemical kinetic to a Hinshelwood-Langmuir-type mechanism.

The overall degradation rate of a solute is the sum of the rates in the bulk and in the interfacial layer. Therefore:

r = Kb + (k × K × Cs)/(1 + K × Cs)(4)

In this equation, r is the degradation rate (mol·L^−1^·min^−1^), and Cs (mol·L^−1^) is the organic compound concentration. Kb (mol·L^−1^·min^−1^) is a constant representing the rate of degradation in the bulk liquid, k is the rate constant of the reaction (mol·L^−1^·min^−1^), and K is the equilibrium constant.

Later, Okitsuet al. [32] developed another model based on the fact that organic molecules adsorb anddesorb from the liquid interface layer surrounding the cavitation bubble, reaching a pseudo-steady state:

r = (k × K × Cs)/(1 + K × Cs)(5)

In this equation, the degradation rate r (mol L^−1^·min^−1^) and the organic compound concentration Cs (M) lead to the determination of the kinetic parameters: k is the rate constant of the reaction (mol L^−1^·min^−1^) and K is the equilibrium constant.

Depending on the water solubility and the HL constant, this model connected to the Hinshelwood-Langmuir model seems appropriate for describing the sonochemical degradation kinetic ofnonvolatile compounds [33].

In the case of BT, the main reactions involved can be summarized in the following way:

Cs = BT concentration in the solution, Ci = BT concentration at the interface.

(6) $$\begin{array}{l} C_{s}\overset{k_{1}}{\underset{k_{2}}{⇌}}C_{i}\\ C_{i}+{}^{{}^{\circ}}\mathrm{OH}\overset{k_{3}}{⟶}P_{\mathrm{ox}} \end{array}$$

Then, from Okitsu et al.’s equation:

- the equilibrium constant K = k_1_/k_2_
- the rate constant k = k_3_[^o^OH]

This model assumes that °OH formation is constant for a reactor and for the fixed ultrasonic power applied, and does not depend on the concentration of the product at the interface: The form of this equation adequately explains why the kinetics of degradation fit well with the “curve fitting” of the Michaelis-Menten kinetic from the KaleidaGraph software.

In the basic Michaelis-Menten model:

r = (r_m_ × Cs)/km + Cs

With r_m_ = maximum rate and km = {k_2_ + (k_3_ × ^o^OH)}/k_1_

The degradation reaction rates and constants from the Michaelis-Menten model can be obtained from the “curvefitting” of the Michaelis-Menten kinetic from the KaleidaGraph software.

Deionized Water: r_DW_ = (11.7 × Cs)/(155.7 + Cs)

Natural Water: r_NW_ = (6.07 × Cs)/(82 + Cs)

Synthetic Sea Water: r_SW_ = (10.7 × Cs)/(205 + Cs)

**Figure 8:**
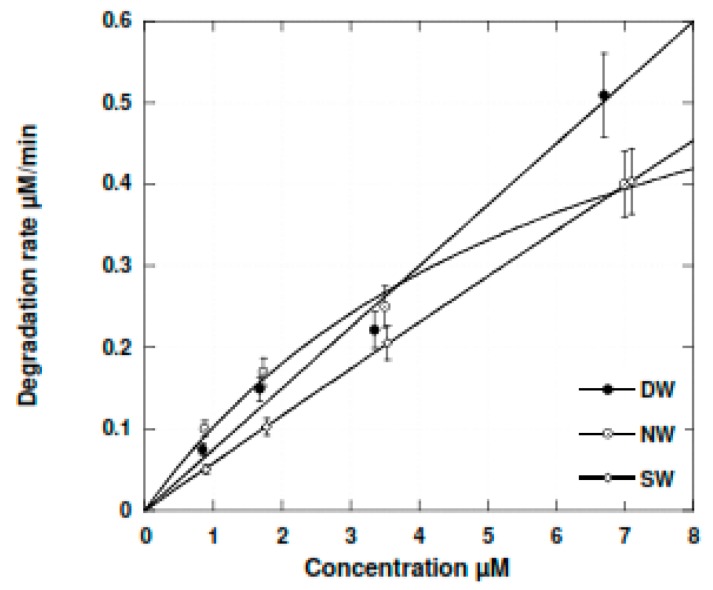
BT degradation rate in deionized, natural and synthetic sea water at the lowest concentrations; 352 kHz; volume = 300 mL; temperature = 21 °C ± 1 °C; electric input = 80 W.

This is shown in the Figure 5 plot, which represents the BT degradation rate in deionized, natural and synthetic water. This plot: r = f_Cs_ is clear evidence that the presence of inorganic salts has a limited detrimental effect onthe sonochemical degradation of BT, mainly in the high concentration range. For the lowest BT concentrations, the presence of less than 5 µM of bicarbonate in NW enhances the degradation rate (Figure 8).

This can occur due to the carbonate and bicarbonate oxidation into the carbonate radical CO_3_^−o^. This radical exhibit lower reactivity than °OH, it has a higher diffusion rate in the bulk of the solution and it can efficiently oxidase aromatic structures [33].

(7) $${\mathrm{HC}O_{3}}^{-}+\;{}^{{}^{\circ}}\mathrm{OH}⟶{{\mathrm{CO}}_{3}}^{-o}+\;H_{2}O\;{CO_{3}}^{2-}+\;{}^{{}^{\circ}}\mathrm{OH}⟶{{\mathrm{CO}}_{3}}^{-o}+\;{\mathrm{HO}}^{-}$$

Attempts to obtain a linear relationship from the determination of rate constants and equilibrium constants from the Okitsu model were unsuccessful.

**Figure 9:**
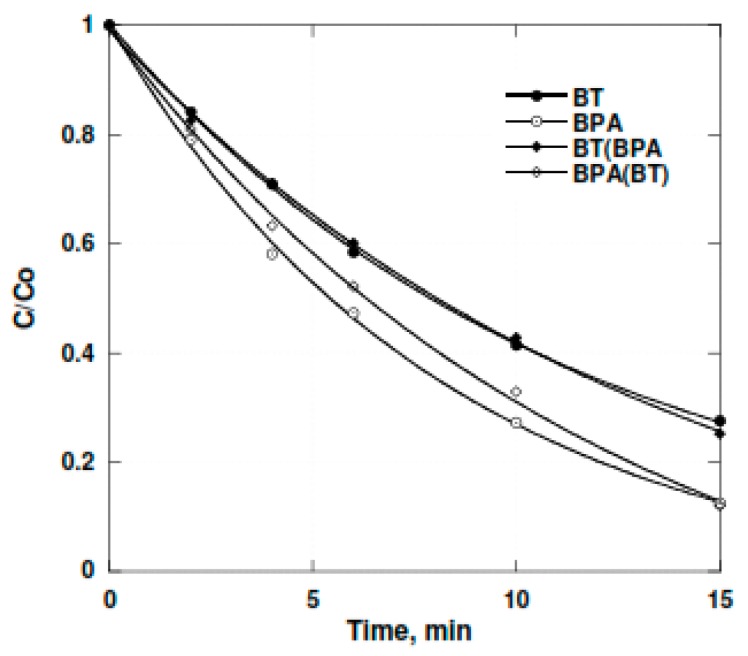
The degradation rate at 352 kHz of a mixture bisphenol A (BPA) (BT-7.0 × 10^−6^ M) in deionized water. Temperature 21 °C ± 1 °C; Electrical input 80 W.

Notably, we extended our investigation to conduct the degradation of BT in the presence of bisphenol A, a compound with a solubility similar to BT and with a higher Henry’s Low constant value than BT (Figure 9).

It was found that even in the presence of BPA, BT degradation was observed. It is interesting to see that BT degradation is unaffected by the presence of BPA. Experiments concerning BT degradation in high concentrations has demonstrated the potential use of the 352 kHz ultrasonic treatment for elimination of the target compounds. This method can be applied in natural and sea water without a noticeable modification to the degradation rates. For the lowest concentrations, the presence of bicarbonate enhances BT removal. 

## 4. Conclusions

The results presented in this work portray the interesting potential that ultrasound has to treat different types of water contaminated with polycyclic aromatic sulfur hydrocarbons (PASHs) such as benzothiophene. The system showed involvement of oxidation rather than the pyrolitic process in the BT sonochemical degradation. In addition to this, when a compound with a solubility similar to BT and a higher Henry’s Low constant value than BT such as BPA is added, it is interesting to see that the BT degradation is unaffected. An ultrasonic generator has the potential for use in environmental decontamination, due to the production of high concentrations of oxidizing species such as ^•^OH and H_2_O_2_ in the solution and localized transient high temperatures and pressures. Furthermore, it does not require the addition of chemical additives to achieve viable degradation rates. Sonochemical processes could be an effective alternative way for the oxidation and complete mineralization of recalcitrant organic compounds. Additionally, by converting the pollutants into less harmful or lower chain compounds, a more efficient biological treatment on the wastewater could be achieved in future work.

## Figures and Tables

**Table 1:** The retention time and molecular ion peaks of BT degradation products observed after 5 min of ultrasonic treatment at 352 kHz for deionized (DW), natural (NW) and synthetic sea waters (SW).

RT	m/z	DW		NW		SW		Assignment
7.98	134	x	x	x	x	x	x	BT (134)
10.80	150	x	x	nd	x	nd	nd	BT + O (134 + 16)
12.10	136	x	x	x	x	x	x	BT + H_2_ (134 + 2)
12.31	150	x	x	x	x	x	x	BT + O (134 + 16)
12.38	150	x	x	x	x	x	x	BT + O (134 + 16)
12.55	150	x	x	x	x	x	x	BT + O (134 + 16)
12.63	150	x	x	x	x	x	x	BT + O (134 + 16)
12.92	166	x	x	x	x	x	nd	BT + 2O (134 + 32)
13.14	150	x	x	x	x	x	x	BT + O (134 + 16)
13.32	166	x	x	x	x	x	x	BT + 2O (134 + 32)
17.16	148	x	x	x	x	x	x	BT + N (134 + 14)
21.09	326	nd	x	nd	x	nd	nd	2BT + 3O (268 + 48)
